# Therapeutic and immunomodulatory activities of short-course treatment of murine visceral leishmaniasis with KALSOME™10, a new liposomal amphotericin B

**DOI:** 10.1186/s12879-015-0928-6

**Published:** 2015-04-17

**Authors:** Mohammad Asad, Pradyot Bhattacharya, Antara Banerjee, Nahid Ali

**Affiliations:** Infectious Diseases and Immunology Division, Indian Institute of Chemical Biology, 4, Raja SC Mullick Road, Jadavpur, Kolkata, 700032 West Bengal India; Present address: Department of Zoology, Bangabasi College, 19 Rajkumar Chakraborty Sarani, Kolkata, 700009 India

**Keywords:** Drug, Liposomal amphotericin B, *Leishmania*, Efficacy, Toxicity, Immunomodulation, Cytokine, Immune response

## Abstract

**Background:**

Visceral leishmaniasis (VL), a potentially fatal disease, is most prevalent in the Indian subcontinent, East Africa and South America. Since the conventional antileishmanial drugs have many limitations we evaluated a new ergosterol rich liposomal amphotericin B formulation, KALSOME™10 for its leishmanicidal efficacy, tolerability and immunomodulatory activity.

**Methods:**

Normal healthy mice were treated with 3.5 mg/kg single and 7.5 mg/kg single and double doses of KALSOME™10. Liver and kidney function tests were performed fourteen days after treatment. Next, normal mice were infected with *Leishmania donovani* amastigotes. Two months post infection they were treated with the above mentioned doses of KALSOME™10 and sacrificed one month after treatment for estimation of parasite burden in the liver and spleen by Limiting Dilution Assay. Leishmanial antigen stimulated splenocyte culture supernatants were collected for cytokine detection through ELISA. Flow cytometric studies were performed on normal animals treated with KALSOME™10, Amphotericin B (AmB) and AmBiosome to compare their immunomodulatory activities.

**Results:**

The drug was found to induce no hepato- or nephrotoxicities at the studied doses. Moreover, at all doses, it led to significant reduction in parasite burden in two month infected BALB/c mice, with 7.5 mg/kg double dose resulting in almost complete clearance of parasites from both liver and spleen. Interestingly, the drug at 7.5 mg/kg double dose could almost completely inhibit the secretion of disease promoting cytokines, IL-10 and TGFβ, and significantly elevate the levels of IFNγ and IL-12, cytokines required for control of the disease. Mice treated with KALSOME™10 showed elevated levels of IFNγ and suppressed IL-10 secretion from both CD4^+^ and CD8^+^ subsets of T cells, as well as from culture supernatants of splenocytes, compared to that of normal, AmB and AmBisome treated animals.

**Conclusions:**

Treatment of infected mice with 7.5 mg/kg double dose of KALSOME™10 was safe and effective in clearing the parasites from the sites of infection. The drug maintains the inherent immunomodulatory activities of AmB by effectively suppressing disease promoting cytokines IL-10 and TGFβ, thereby boosting IL-12 and IFNγ levels. This emphasizes KALSOME™10 as a promising drug alternative for lifelong protection from VL.

## Background

Visceral leishmaniasis (VL) or kala-azar, a disseminated infection of the lymphoreticular system, is caused by the protozoan parasite(s) *Leishmania donovani* and/or *L. infantum/chagasi*. An estimate of 0.2-0.4 million global VL cases are reported each year with more than 90% of them occurring in India, Bangladesh, Sudan, South Sudan, Brazil and Ethiopia [1] Human infections can be asymptomatic or oligosymptomatic with manifestations including persistent low grade fever, hepatosplenomegaly, cachexia, pancytopenia and hypergammaglobulinemia. During active VL the parasites multiply within the macrophages of spleen, liver and bone marrow resulting in fatal outcome if untreated [2]. The course of disease progression signifies cell-mediated immune response (CMI) to play an important role in protection or development of VL [3]. Active VL is characterized by suppression of CMI, which is apparent from the unresponsiveness of patients to different delayed type hypersensitivity (DTH) tests (Leishmanin skin test or Montenegro test) as well as defective lymphoproliferative response of the peripheral blood mononuclear cells [4]. Recovery from infection following an effective chemotherapy, on the other hand, is associated with a strong cell mediated DTH response [5]. Thus a favorable CMI in response to appropriate treatment marks the successful cure of VL.

In the absence of a vaccine, chemotherapy is the mainstay to combat the disease. Until the year 2000, pentavalent antimonials (Sbv) were the frontline treatment for VL. However, with the emergence of antimony resistant parasites in parts of northern Bihar, India, amphotericin B (AmB) was introduced which renders high cure rates (>95%) in VL patients [6]. However, AmB which requires intravenous administration of long duration is highly toxic and has frequent adverse effects, including infusion-related fever and chills, nephrotoxicity, hepatotoxicity and hypokalemia [7]. To overcome its drawbacks lipid formulations of AmB (L-AmB) have been developed to reduce organ toxicity as well as treatment duration. Most of these formulations are, however, exorbitantly costly. Although WHO has recently reduced the price of one such formulation, AmBisome, the cost of these L-AmBs still precludes their widespread use in the developing countries [8]. In this context an Indian formulation of L-AmB, Fungisome has been introduced. A clinical trial with this drug at doses of 2 mg/kg for 10 days, 3 mg/kg for 5 days and 3 mg/kg for 7 days yielded cure rates of 100%, 90.9% and 100% respectively [9]. These encouraging results paved the way for a short-course therapy to determine whether a relatively larger amount of Fungisome could be safely and effectively administered at single (5 and 7.5 mg/kg) or double (5 mg/kg) doses. The treatment regimens manifested a successful cure rate of 50-90% at 6 months posttreatment. A close look at the immunological profile of the VL patients at one week after treatment revealed a significant fall in plasma IL-10 levels in all successfully cured patients. Investigations on the antigen-specific cytokine production by PBMCs showed an enhanced Th1 type response with upregulated IFNγ, IL-12 and TNFα and reduced IL-10 and TGFβ production one week posttreatment in patients who were successfully cured at 6 months irrespective of the drug-dose [10]. These results with Fungisome could match the immunological profile obtained with AmB as early as one week posttreatment demonstrating the importance of immunomodulatory effects exerted by the disease inhibiting cytokines for successful cure [7,10].

Most of the L-AmBs in clinical use contain cholesterol as one of the constituents and is useful for stability and targeted delivery of the drug [9,11,12]. Cholesterol, on the other hand, plays a crucial role in active VL facilitating the internalization of the parasites [13]. Recent work of Chandel *et al.* demonstrates that exogenous cholesterol, if added in the culture, can enhance the growth of *Leishmania* promastigotes [14]. Therefore, after internalization, the cholesterol requirement by *Leishmania* for its sustenance may be fulfilled by salvaging cholesterol from host macrophages. Hence, formulation of liposomal AmB devoid of cholesterol is a good strategy in designing antileishmanial drugs. Thus, new amphiphilic L-AmB, KALSOME™10 has been developed where AmB is intercalated with sterol and phosphatidyl choline. This is a sterol rich drug where ergosterol constitutes 50% molarity of total lipid in the liposome [15]. Absence of cholesterol could make this drug more suitable for clearing parasites. A recent study with KALSOME™10, at 7.5 mg/kg triple dose, reported successful therapy (98.85% amastigote suppression) of BALB/c mice with established *L. donovani* infection [16]. Here we have evaluated lower doses of KALSOME™10 (3.5 mg/kg single dose, 7.5 mg/kg single dose and 7.5 mg/kg double dose) for their efficacy in curing murine VL. In addition, we were interested to investigate whether a similar modulation of cytokine profile as observed with AmB treatment could be obtained with KALSOME™10 to identify the possible mechanism of cure. Along with efficacy, tolerability of these doses was also evaluated through different liver (SGOT, SGPT and alkaline phosphatase) and kidney (urea and creatinine) functioning parameters.

## Methods

### Drug, kits and chemical reagents

KALSOME™10 (formulation code: K10, concentration: 10 mg/ml) was provided by Lifecare Innovations Pvt. Ltd., Gurgaon, India. Toxicity study kits (SGPT, SGOT, Alkaline phosphatase, Urea and Creatinine) were purchased from Randox Molecular Diagnostics, London, UK. ELISA kits for cytokines IL-10, IL-12 and IFNγ were obtained from BD Biosciences (San Diego, CA, USA) and TGFβ from eBioscience (San Diego, CA, USA). The following fluorochrome tagged mAbs obtained from BD Pharmingen were used: anti-mouse CD4 PE-Cy7, anti-mouse CD8a Percp-Cy5.5, IFNγ Alexa fluor 488 and IL-10 APC.

### Animals and parasites

BALB/c mice (4–6 weeks old) used in the experiments were bred in the Institute’s animal house facility. The studies were performed according to the Committee for the Purpose of Control and Supervision on Experimental Animals (CPCSEA), Ministry of Environment and Forest, Govt. of India, and approved by the animal ethics committee (147/1999/CPSCEA) of Indian Institute of Chemical Biology. *L. donovani* strain AG83 (MHOM/IN/1983/AG83), originally isolated from an Indian kala-azar patient, was maintained by serial passage in hamsters [17].

### Formulation of KALSOME™10

KALSOME™10, a gift from Lifecare Innovations Pvt Ltd, India is a sterol enriched liposome, intercalating AmB, in an aqueous suspension. Ergosterol constitutes almost 50% of the total liposomal lipid, overall consisting of phospatidylcholine, ergosterol and AmB in 5:2:1.8 ratios. The composition was sonicated during manufacture and before administration for increasing plasma half-life and better bio-distribution [15].

### In vivo efficacy study

**Figure 1:**
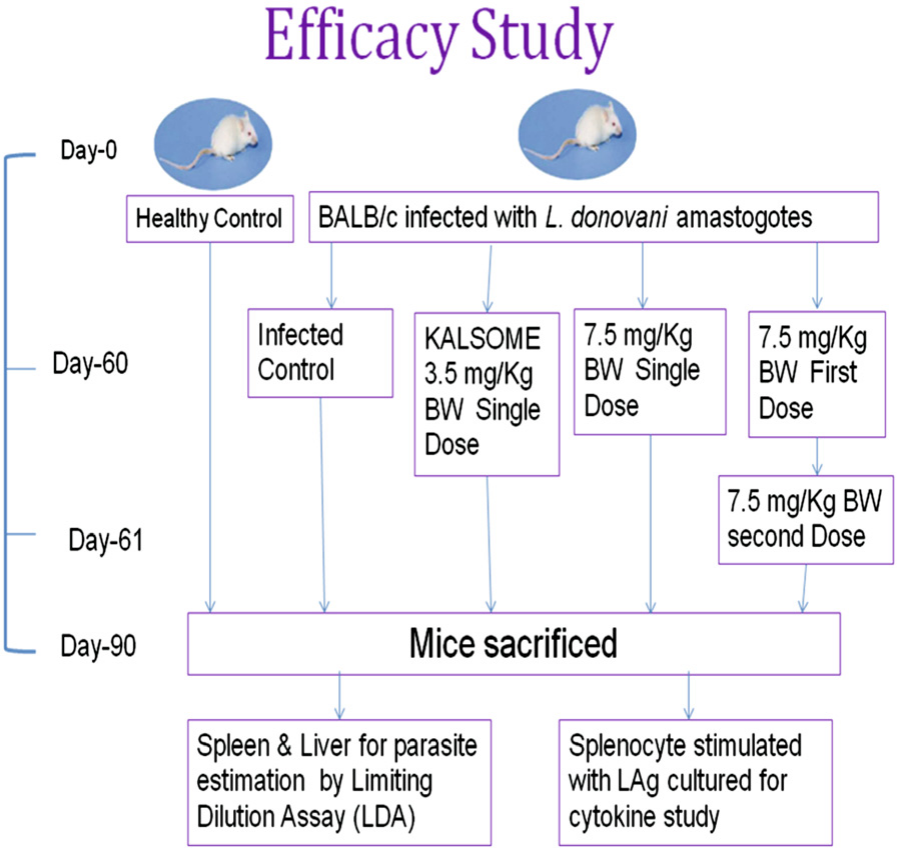
Plan of work for efficacy study of KALSOME™10.

Thirty healthy BALB/c mice (4–6 weeks old) were infected with 2.5 × 10^7^ freshly isolated amastigotes [18]. Six mice were randomly sacrificed after two months to estimate the parasite burden (liver LDA = 10.65 ± 0.92 and spleen LDA = 8.57 ± 0.54). The rest were divided randomly into four different experimental groups. One group was kept as infected control and others were administered with 3.5 mg/kg and 7.5 mg/kg single doses and 7.5 mg/kg double dose of KALSOME™10 respectively dissolved in 200 μl of 0.02 M PBS through the tail vein (Figure 1).

### Determination of parasite burden

One month after treatment all the mice were sacrificed, spleen and liver were removed aseptically, and parasite burden of the organs were quantified by Limiting Dilution Assay (LDA) as described earlier [18]. Briefly weighed pieces of spleen and liver from experimental mice were first homogenized in Schneider’s medium supplemented with 10% FCS, and then diluted to a final concentration of 1 mg/ml. Five-fold serial dilutions of the homogenized tissue suspension were plated in 96-well plates and incubated at 22°C. Wells were examined for viable and motile promastigotes after 15 days and the reciprocal of the highest dilution positive for parasites was considered as the parasite concentration per mg of tissue. The total organ parasite burden was calculated using the weight of the respective organs. In a separate study BALB/c mice were infected similarly for two months and treated with AmB and AmBisome with 2.5 and 3.5 mg/kg AmB respectively. LDA of liver and spleen was performed as described above.

### In vivo toxicity study

**Figure 2:**
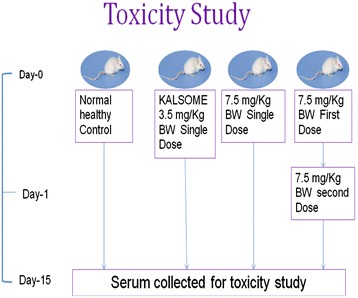
Plan of work for toxicity study of KALSOME™10.

For toxicity study, twenty four healthy adult (8–10 weeks old) BALB/c mice weighing approximately 25 g were randomly divided into groups of six mice each. One group was kept as control and rest were injected with 3.5 mg/kg single dose, 7.5 mg/kg single dose and 7.5 mg/kg double dose of KALSOME™10, respectively, in 200 μl of 0.02 M PBS through the tail vein. Liver (SGPT, SGOT and alkaline phosphatase) and kidney (urea and creatinine) toxicity tests were performed 14 days after drug administration [19] and compared with that of the control group (Figure 2).

### Preparation of leishmanial promastigote membrane antigen (LAg)

LAg was prepared from *L. donovani* promastigotes as described earlier [18]. Briefly, stationary phase promastigotes were washed in PBS (pH 7.2) and resuspended in 5 mM Tris HCl buffer (pH 7.6). The suspension was vortexed and centrifuged at 2,310 g for 10 min. The crude ghost membrane pellet was resuspended in the same buffer and sonicated for 3 min by an ultrasound probe sonicator (Misonix, Farmingdale, NY). The suspension was centrifuged at 5,910 g for 30 min and the supernatant containing LAg was stored at −80°C until use. The amount of protein obtained from a 1.0 g cell pellet was approximately 24 mg.

### Cell proliferation and cytokine assays

For cell proliferation and cytokine assay, spleens were aseptically removed from normal, infected and treated animals one month posttreatment. Single cell suspension was prepared in RPMI 1640 supplemented with 10% FBS, l00 U/ml penicillin G sodium, 100 μg/ml streptomycin sulfate and 50 μM β-mercaptoethanol. RBCs were removed by lysis with 0.14 M Tris buffered NH_4_Cl. The remaining cells were washed twice in culture medium and viable mononuclear cell number determined by counting Trypan blue unstained cells in a hemocytometer. The cells (1 × 10^6^/ml) were then cultured in duplicate in 96-well flat-bottom tissue culture plates (Nunc) and stimulated with LAg (10 μg/ml) for 3 days at 37°C in 95% humidified air with 5% CO_2_ [7]. To compare the immunomodulatory activity of KALSOME™10 with that of AmB and AmBisome, normal BALB/c mice were divided into eight groups. Group I was kept as healthy control. Groups II, III and IV were administrated with 3.5 mg/kg single dose, 7.5 mg/kg single dose and 7.5 mg/kg double dose of, KALSOME™10, respectively. Group V was given 2.5 mg/kg AmB and groups VI, VII and VIII were administrated with 3.5 mg/kg single dose, 7.5 mg/kg single dose and 7.5 mg/kg double dose of, AmBisome, respectively in 200 μl of 0.02 M PBS through the tail vein. Splenocyte cultures from normal and treated groups of mice were stimulated with ConA (2.5 μg/ml). For cytokine analysis, culture supernatants were collected after 72 h and concentrations of IFNγ, IL-12, TGFβ and IL-10 were estimated by ELISA according to the manufacturer’s instructions.

### Flow cytometry

Flow cytometry was performed on various T cell subsets and the intracellular cytokines produced by these cells from normal and drug treated mice were analyzed as described [18]. Splenocytes from different groups of experimental and healthy mice were stimulated for 12 hours with ConA (2.5 μg/ml). Brefeldin A (10 μg/ml) was added to the cultures 2 hours before harvesting. The cells were then washed in FACS buffer (0.02 M PBS and 1% FBS) and stained with fluorescent conjugated surface markers for CD3, CD4 and CD8. Subsequently, cells were permeabilized with FACS permeabilizing solution and washed in FACS buffer containing 0.1% saponin. Then the cells were stained for cytokines IL-10 and IFNγ and data were acquired by LSRFortessa flow cytometer (BD Biosciences) and analyzed through FACS Diva software (BD Biosciences).

### Statistical analysis

All data comparisons were tested for significance by One-way analysis of variance (ANOVA) and Tukey’s multiple comparisons using Graph Pad Prism version 5.0 (Graphpad Software, v. 5.0, San Diego, CA). Results with *p* < 0.05 were considered to be statistically significant.

## Results

### Effective clearance of *Leishmania* parasite by KALSOME™ 10

**Figure 3:**
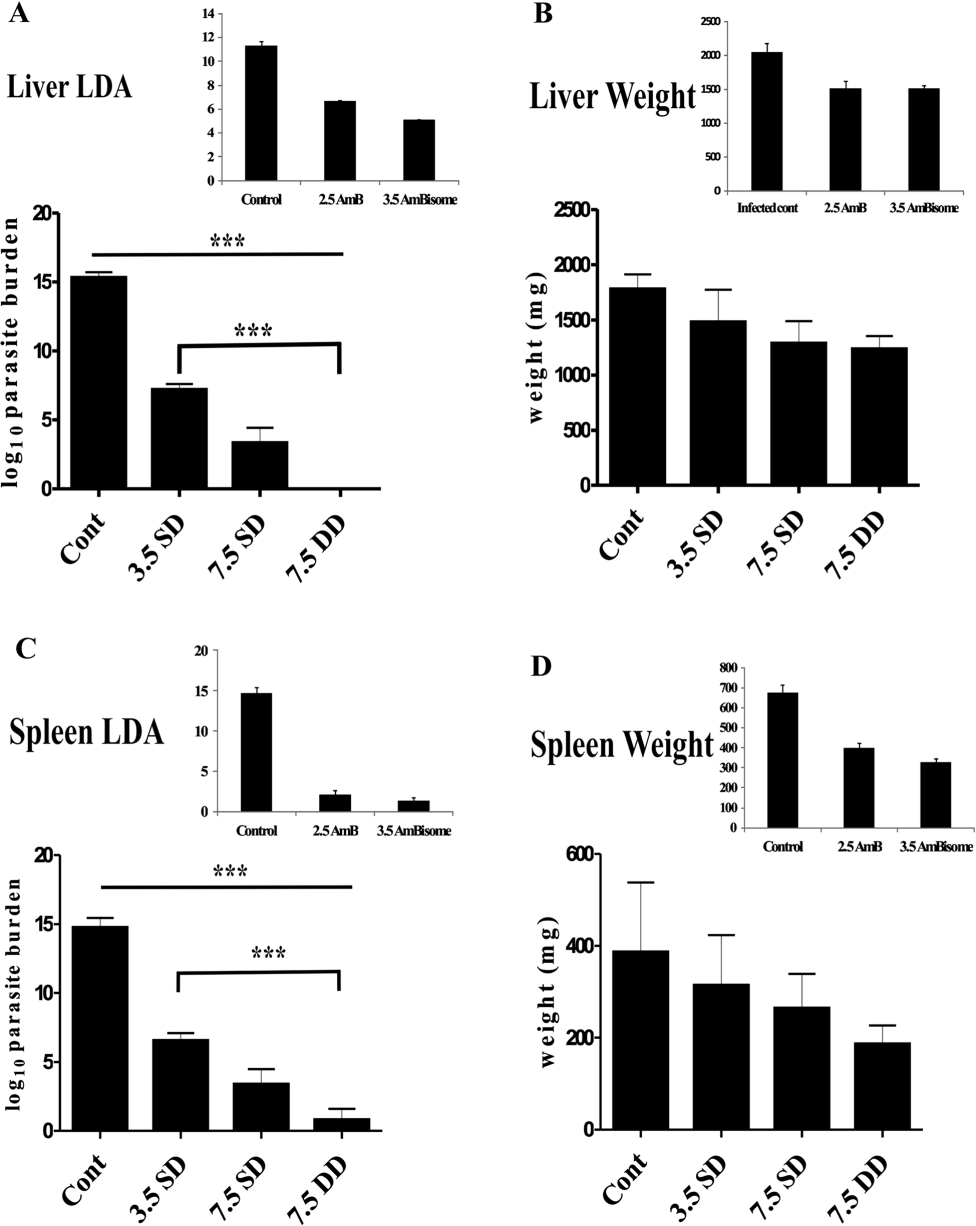
Efficacy of KALSOME™10 in murine VL. Two month infected animals were treated with 3.5 mg/kg single dose (SD) and 7.5 mg/kg SD and double doses (DD) of KALSOME™10 in 200 μl of 0.02 M PBS through the tail vein. One month post treatment, LDA was performed for evaluating their efficacy. Parasite burdens **(A and** **C)** and weights **(B and** **D)** of liver and spleen show the changes at different doses with normal and infected mice. Data expressed as means ± SE for six mice per group and are representative of two independent experiments with similar results. ***P < 0.001.

To estimate the efficacy, low doses of KALSOME™ 10 (3.5 mg/kg single dose, 7.5 mg/kg single dose and 7.5 mg/kg double dose) were injected into 2 months infected BALB/c mice. One month after treatment LDA was performed to evaluate parasite burden at the sites of infection *i.e.* liver and spleen. Treatment with 3.5 mg/kg and 7.5 mg/kg single doses of KALSOME™ 10 showed a substantial fall (~3-fold) in parasite levels in these organs compared to infected controls. Administration of 7.5 mg/kg double dose, on the other hand, resulted in almost complete clearance of the parasites from both liver and spleen (Figure 3A and C). Although all the drug doses were significantly effective in clearing the parasites from liver and spleen (*P* < 0.001), 7.5 mg/kg double dose appeared to be most effective with parasite clearance efficiency significantly higher (*P* < 0.001) than even 3.5 mg/kg single dose. The weights of infected liver and spleen also gradually decreased with increase in drug doses (Figure 3B and D) when compared with infected controls, although the difference was not significant. Moreover, the efficacy of KALSOME™ 10 was comparable to that of AmB and AmBisome (Figure 3A and C). Treatment of infected mice with 3.5 mg/kg single dose of KALSOME™ 10 generated similar efficacy as that with a single dose of 3.5 mg/kg AmBisome and 2.5 mg/kg AmB when compared in both liver and spleen.

### *In vivo* toxicity study of effective dose of KALSOME™ 10 in non-challenged BALB/c mice

**Figure 4:**
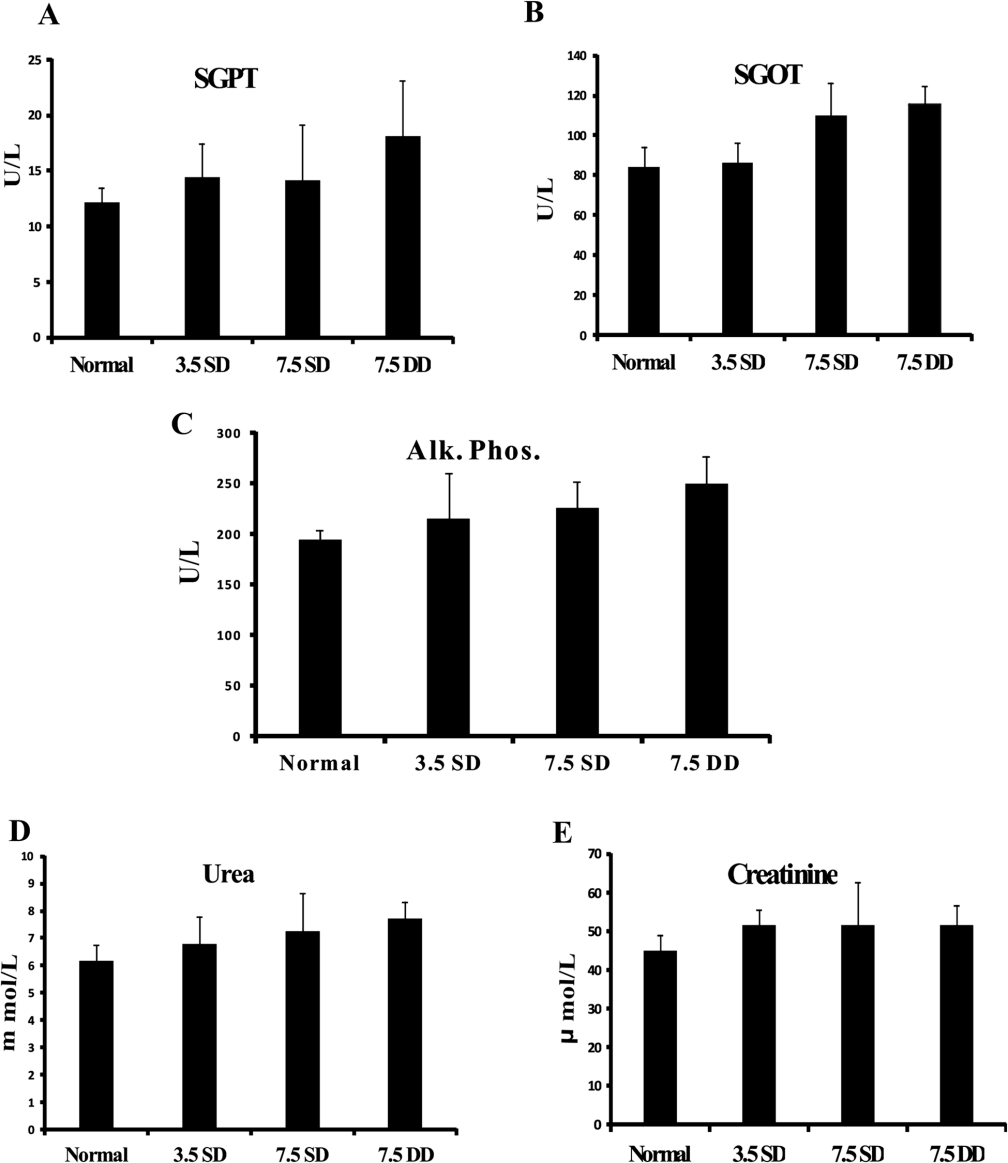
Toxicity study of KALSOME™10 in normal mice. Healthy BALB/c mice were treated with 3.5 mg/kg SD and 7.5 mg/kg SD and DDs of KALSOME™10 in 200 μl of 0.02 M PBS through the tail vein. Fourteen days post treatment, liver [SGPT **(A)**, SGOT **(B)** and Alkaline Phosphatase **(C)**] and kidney [urea **(D)** and creatinine **(E)**] functioning tests were performed on mice serum and compared with that of healthy controls. Data represented as means ± SE for six mice per group and are representative of two independent experiments with similar results.

To detect any functional abnormality of liver due to KALSOME™ 10 treatment, the drug was administered into normal healthy BALB/c mice at three different doses (3.5 mg/kg single dose, 7.5 mg/kg single dose and 7.5 mg/kg double dose) and SGPT, SGOT and alkaline phosphatase levels measured at 14 days posttreatment. Treatment with single drug doses of 3.5 mg/kg and 7.5 mg/kg resulted in comparable levels of SGPT and alkaline phosphatase whereas administration of 7.5 mg/kg double dose exhibited minor increase in these parameters compared to untreated controls (Figure 4A and C). Levels of SGOT in mice treated with 3.5 mg/kg single dose remained comparable to normal values (Figure 4B). However, 7.5 mg/kg single dose and double dose treated mice exhibited some rise in SGOT levels compared to controls. These differences, however, were insignificant indicating no aberration in liver function due to drug administration. Along with hepatotoxicity, nephrotoxicity is also a major challenge for a prospective drug candidate against leishmaniasis. The mean level of creatinine for all the drug doses was found to be similar (Figure 4E) with a small but insignificant increase in urea levels at 7.5 mg/kg single and double doses when compared to untreated controls (Figure 4D).

### KALSOME™ 10 mounts a protective response against VL by regulating different disease promoting/inhibiting cytokines

**Figure 5:**
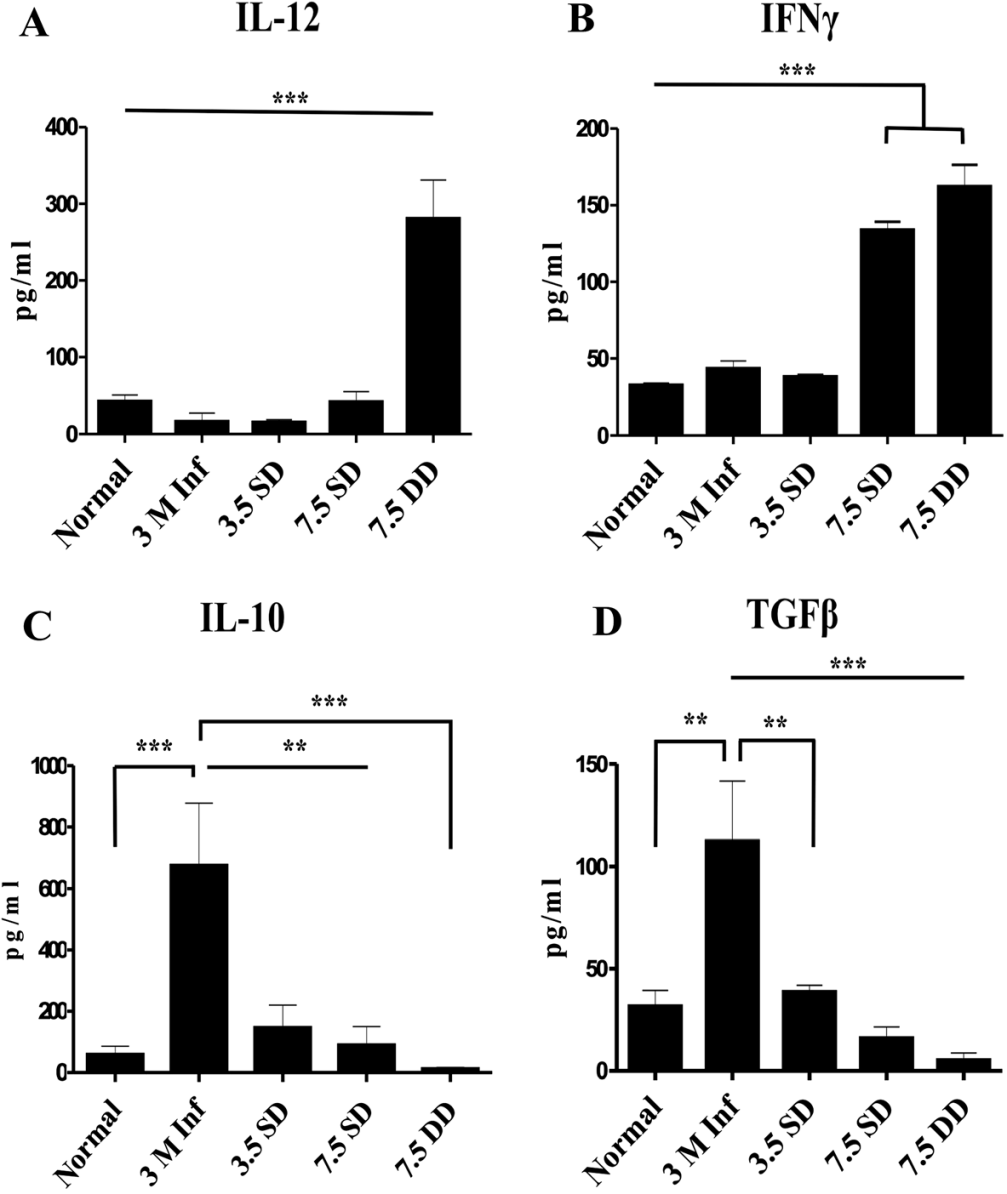
Immunomodulatory role of KALSOME™10. Two month infected BALB/c mice were treated with 3.5 mg/kg SD and 7.5 mg/kg SD and DDs of KALSOME™10. Leishmanial antigen stimulated culture supernatants of splenocytes obtained from normal, infected and treated mice were used for cytokine profiling [IL-12 **(A)**, IFN-γ **(B)**, IL-10 **(C)** and TGFβ **(D)**]. Data represented as means ± SE for six mice per group and are representative of two independent experiments with similar results. *P < 0.05, **P < 0.01, ***P < 0.001.

VL is associated with impaired cell mediated immunity marked by the inability of T cells to proliferate or to produce IFNγ in response to leishmanial antigens [20]. Recent investigations have reported the ability of these cells to respond to leishmanial antigens with the production IL-10 and TGFβ, the key disease promoting factors in VL [7,21]. The development of resistance and control over parasites require the production of IL-12 from antigen presenting cells and IFNγ from T cells [21]. To investigate whether KALSOME™ 10 therapy can modulate the disease promoting immune response to effective immunity, we carried out a detailed immunoprofiling of leishmanial antigen stimulated splenocyte culture supernatants of normal, infected and treated mice. Three month infected BALB/c mice demonstrated significantly higher levels of IL-10 (*P* < 0.001) and TGFβ (*P* < 0.01) in comparison to normal mice, whereas there was no change in the levels of IL-12 and IFNγ when compared to normal controls (Figure 5A and B). Treatment with KALSOME™ 10 at 3.5 mg/kg single, 7.5 mg/kg single and double doses exhibited varying curing efficacies ranging from 53-100% (Table 1). Interestingly, however, significant fall in the levels of IL-10 (*P* < 0.01) and TGFβ (*P* < 0.01) was observed in all the groups in comparison to infected mice. Nevertheless, 7.5 mg/kg double dose, which showed the most effective cure, resulted in almost complete inhibition of both of IL-10 and TGFβ (Figure 5C and D). Again, KALSOME™ 10 induced significantly (*P* < 0.001) higher levels of IL-12 and IFNγ at 7.5 mg/kg double dose (Figure 5A and B) with 7.5 mg/kg single dose also promoting significant (*P* < 0.001) elevation of IFNγ (Figure 5B) emphasizing immunomodulation from disease promoting cytokine milieu to a strong IL-12 and IFNγ secretion for effective cure against VL.

**Table 1:** **Percent reduction in parasite burden by KALSOME™** **10, AmB and AmBisome therapy, estimated by LDA**

**Drug**		**Percent reduction in LDA values**	
		**Liver**	**Spleen**
3.5 mg/kg SD*	KALSOME™10	53.02	55.7
7.5 mg/kg SD	KALSOME™10	78.1	77.02
7.5 mg/kg DD	KALSOME™10	100	94.6
2.5 mg/kg	AmB	40.65	57.39
3.5 mg/kg	AmBisome	54.78	72.02

Groups of 3–6 infected BALB/c mice were treated with the various doses of the drugs (KALSOME™10, AmB and AmBisome). After one month, mice were sacrificed to estimate the parasite burden as described in Methods. Percent reduction in LDA of liver and spleen was calculated based on that of infected control. *SD: single Dose; ^¶^DD: Double Dose.

### KALSOME™ 10 treatment induced protective immunity correlates with a protective cytokine milieu

**Figure 6:**
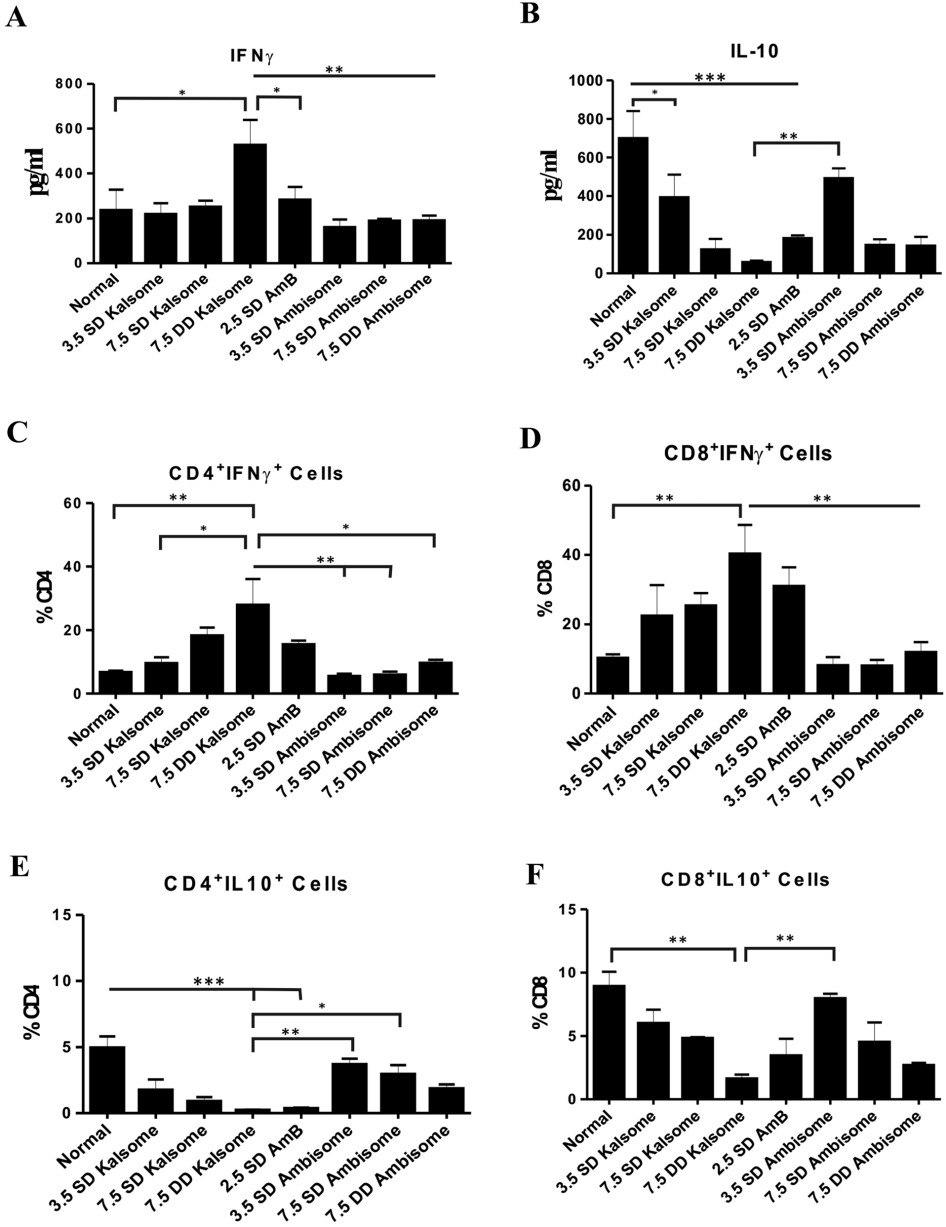
Immunomodulatory role of KALSOME™10 on prophylactic model. Normal healthy mice were treated with different doses of KALSOME™10 (3.5 mg/kg single, 7.5 mg/kg single and double), AmBisome (3.5 mg/kg single, 7.5 mg/kg single and double) and AmB (2.5 mg/kg) in seven different groups with four mice in each group. One group was kept as normal healthy control. Spleen cells of differently treated animals were isolated 10 days after treatment, plated aseptically (2.5 × 10^5^ cells/well), and stimulated with Con A (2.5 μg/ml). Levels of ConA-specific IFNγ **(A)** and IL-10 **(B)** were determined after 72 hours of incubation by ELISA. Mean frequencies of CD4^+^IFNγ^+^ cells per total CD4^+^ cells **(C)**, CD8^+^IFNγ^+^ cells per total CD8^+^ cells, **(D)** CD4^+^IL-10^+^ cells per total CD4^+^ cells **(E)** and CD8^+^IL-10^+^ cells per total CD8^+^ cells **(F)** of ConA stimulated splenocytes were determined by flow cytometry after 12 hours of incubation. Data represented as means ± SE for four mice per group and are representative of two independent experiments with similar results. *P < 0.05, **P < 0.01, ***P < 0.001.

To compare the immunomodulatory effects of KALSOME™10 with AmB and AmBisome, we administered 3.5 and 7.5 mg/kg (single and double) doses of KALSOME™10 and AmBisome in groups of normal mice. Another group was injected with 2.5 mg/kg dose of AmB, the maximum tolerable dose of this drug, keeping an untreated group as control. After 10 days the animals were sacrificed and ConA stimulated splenocytes were cultured for cytokine ELISA and FACS analysis. In cytokine ELISA study, we found that IFNγ secretion was significantly high from 7.5 mg/kg double dose of KALSOME™10 treated mice compared to all other groups (Figure 6A). This elevated secretion of IFNγ correlated well with the suppression of IL-10 production by this dose. Similarly, significantly high frequencies of CD4^+^ and CD8^+^ cells producing IFNγ were observed by 7.5 mg/kg double dose of KALSOME™10 compared to 2.5 mg/kg AmB and all administered doses of AmBisome (Figure 6C and D). Although IFNγ production from CD8^+^ cells was significantly high in AmB treated group, the highest production of IFNγ was still observed by 7.5 mg/kg double dose KALSOME™10 treatment. Significant suppression of IL-10 production was observed in 2.5 mg/kg dose of AmB and 7.5 mg/kg double dose of AmBisome (Figure 6B), suppression of IL-10 production by CD4^+^ and CD8^+^ T cells in 7.5 mg/kg double dose KALSOME™10 treated group was most prominent compared to normal (Figure 6E and F). Moreover, lowest level of IL-10 was detected from CD4^+^ cells of the same group, reaching almost baseline.

## Discussion

AmB, a polyene macrolide, has emerged as the main antileishmanial drug following the prevalence of SAG resistant *Leishmania* strains in India. This drug has proved to be very effective against VL with a cure rate of almost 100% [22]. However, the toxic effects associated with AmB administration limits its utility [23]. Several lipid formulations of AmB especially AmBisome have been exploited very effectively for reducing its toxic effects and targeted delivery of AmB against *Leishmania* infection [24,25]. Unfortunately these formulations are costly and imported, thereby further increasing the treatment cost. Furthermore, recent reports of VL relapses and development of PKDL after apparent cure with AmBisome [26,27] demonstrate the need to develop new L-AmBs. In addition to its antileishmanial activities, AmB has been found to possess immunomodulatory function. Therapy with AmB induced elevated production of TNFα, IFNγ and IL-12 in splenocytes of treated mice, and PBMC of kala-azar patients with reduced IL-4, IL-10 and TGFβ production [7,18]. Interestingly AmB could upregulate IFNγ as well as suppress IL-10 and TGFβ in normal mice splenocytes and human PBMCs suggesting its inherent immunomodulatory activity [7]. AmBisome, on the contrary, could neither promote Th1 response nor downregulate Th2 cytokines [18] suggesting a lack in immunomodulatory function in this L-AmB, whereas essential for parasite clearance and prevention of relapse following drug treatment [7]. Therefore the need for an efficient and cost effective liposomal AmB, for short course treatment of leishmaniasis, having long-lasting protective effect remains. Herein we investigated the curative efficacy and immunomodulatory activity of a new liposomal formulation of AmB, KALSOME™10 at single (3.5 mg/kg and 7.5 mg/kg) and double dose (7.5 mg/kg) therapy. While all the doses led to significant reduction in the parasite burden in the two month infected BALB/c mice, treatment with 7.5 mg/kg double dose led to almost complete cure in both liver and spleen. These doses were found to be safe with no hepatic and renal impairment. Further, as observed in the therapy with free AmB [7], KALSOME™10 maintained the inherent immunomodulatory efficacy of the AmB and augmented Th1 immune response by suppressing the disease promoting cytokines, IL-10 and TGFβ.

*Leishmania* is unique in having the ability to survive and multiply within host neutrophils and macrophages. AmB promotes leishmanicidal activity by virtue of its high intercalation affinity for ergosterol or its precursor present on the parasite [28]. It, however, can also interact with the cholesterol present in host cell membrane inflicting toxicity and functional impairment of the reticuloendothelial system (RES) [29]. In KALSOME™10, AmB is intercalated with ergosterol which constitutes 50% molarity of the total lipid of the liposome [15]. Mechanism of antileishmanial activity of AmB is based on its interaction with the sterols present on parasite cell membrane and the subsequent parasiticidal activity. There are two sterols of relevance while designing a drug to treat *Leishmania* infection. One of them is cholesterol, present mostly in human kidney cell membranes and the other is ergosterol, present in parasite. It is noticeable that affinity of AmB for ergosterol is aproximatly 8.5 times more than that of cholesterol [30]. For effective treatment, targeted delivery of high doses of AmB is the desired strategy. Encapsulating higher amount of AmB in cholesterol containing liposomes may result in leakage of AmB from the liposomes to the human tissues and organs. To prevent this cholesterol has been replaced by ergosterol. This strategy ensures that AmB will not be released from liposomes until it reaches the target. The breaking up of the liposomes occurs inside the macrophages, residence of *Leishmania*. Thus ergosterol encapsulated AmB has a definite edge over cholesterol encapsulated AmB due to its slow release and greater target specificity. Also due to higher affinity of AmB towards ergosterol than cholesterol, greater amount of AmB can be tightly packed inside the liposome and it can be targeted to the macrophages more efficiently [15]. In our study we revaluated the tolerability of KALSOME™10 at 3.5 mg/kg, 7.5 mg/kg single and double doses by assessing different nephro- and hepatotoxic parameters and found it to be non-toxic at all of these doses. Since L-AmBs are not entirely free of infusion related toxicities [22,31] and AmBisome has been reported to show nephrotoxicity at 5 mg/kg triple dose [22,32] these results were very encouraging and could provide a new antileishmanial treatment option.

The parasiticidal efficacy of KALSOME™10, at 7.5 mg/kg triple dose, has already been established in *L. donovani* infected BALB/c mice [16]. Our interest was to determine whether it could successfully maintain its leishmanicidal activity at lower doses (3.5 mg/kg single dose, 7.5 mg/kg single and double doses) lower than the reported one. Herein we showed that therapy of established murine VL with increasing doses of KALSOME™10 led to progressive reduction in the parasite load in the visceral organs with 7.5 mg/kg double dose exhibiting complete parasite clearance in both liver and spleen. Additionally reduction in parasite load with increasing drug doses correlated with the decrease in the organ weight synchronizing with progressive cure. The parasite killing efficiency achieved by KALSOME™10 at such low doses could be matched only by very few antileishmanial drugs [33]. While 5 mg/kg triple dose of AmBisome showed similar efficacy in murine VL, it required longer treatment duration [34]. Even in kala-azar patients a similar dose (5 mg/kg/day for 3 days in a span of 10 days for a total dose of 15 mg/kg) of AmBisome was reported to cure only 90% of the patients with few relapses [35,36]. Thus KALSOME™10 has the potential to become a successful treatment alternative to the presently used antileishmanial chemotherapies at doses that are safe and tolerable.

It is well established that the course of disease progression following *L. donovani* infection is modulated by a range of T cell responses and cytokine network. Extensive studies have identified IL-10 as the major player in the disease pathology of VL [3]. It can render macrophages unresponsive to activation signals and inhibit killing of amastigotes by downregulating the production of NO [22]. TGFβ has also been reported to have inhibitory effects on the action of macrophages and its blockade has been found to limit parasite replication in host cells [21]. Effective elimination of IL-10 and TGFβ synchronizing with the upregulation of IFNγ and IL-12 could be key factors for therapeutic success against VL [7,37,38]. It has been reported that chemotherapy with SAG and AmB cause downregulation of disease promoting cytokines IL-10 and TGFβ, leading to enhanced IL-12 and IFNγ levels correlating with cure [5,7,39]. Herein we also found that treatment of infected mice with increasing doses of KALSOME™10 (3.5 mg/kg single dose and 7.5 mg/kg single and double doses) correlated with the reduction of both the disease promoting cytokines, IL-10 and TGFβ. Moreover, IL-10 was completely absent and secretion of TGFβ was almost negligible at 7.5 mg/kg double dose of KALSOME™10. Subsequently we observed elevation in both IL-12 and IFNγ levels in infected mice treated with 7.5 mg/kg double dose. Treatment with 7.5 mg/kg double dose effectively suppresses disease promoting cytokines IL-10 and TGFβ, thereby boosting IL-12 and IFNγ levels. This immune modulation by KALSOME™10 may be responsible for the almost complete parasite clearance observed by 7.5 mg/kg double dose.

Notably KALSOME™10 showed marked immunomodulatory effect at single and double doses of 7.5 mg/kg by significantly increasing IFNγ level leading to suppression of IL-10. Elevated production of IFNγ from CD4^+^ and CD8^+^ T cells in 7.5 mg/kg KALSOME™10 treated groups led to maximum suppression of IL-10 production from these cells. Such an elevation of IFNγ was almost absent in AmBisome at all comparative doses. Although some immunomodulatory activity was detected by AmB treatment, the best results were observed only with KALSOME™10. Therefore, encapsulation of AmB in our formulation not only maintains the inherent immunomodulatory effects of AmB, but also enhances it, which is a prerequisite for long term protection.

## Conclusion

In conclusion, we can say that treatment of *L. donovani* infected mice with KALSOME™10 at 7.5 mg/kg double dose resulted in almost complete parasite clearance from both liver and spleen without inflicting any liver and kidney toxicity. The decline in parasite number was supported by a curative immune response with a significant fall in disease promoting cytokines (IL-10 and TGFβ) and subsequent increase in protective cytokines (IL-12 and IFNγ). These results with KALSOME™10 at 7.5 mg/kg double dose could match the immunological profile obtained with AmB as early as two days posttreatment without showing any infusion related side effects. However, the role of KALSOME™10 in prevention of reinfection after cure and long term protection from VL need further investigation. Till date KALSOME™10 has only been used in experimental animals, but when it is marketed its price is expected to be 25% less than AmBisome by virtue of its 10 times more AmB associated with liposome in comparison to AmBisome.
